# Pain Symptoms in Fibromyalgia Patients with and without Provoked Vulvodynia

**DOI:** 10.1155/2014/457618

**Published:** 2014-01-29

**Authors:** Anna Ghizzani, Valentina Di Sabatino, Anna Lisa Suman, Giovanni Biasi, Enrica Laura Santarcangelo, Giancarlo Carli

**Affiliations:** ^1^Department of Molecular Medicine and Development, Siena University Hospital, 53100 Siena, Italy; ^2^Rheumatology Clinic, Siena University Hospital, 53100 Siena, Italy; ^3^Department of Medicine, Surgery and Neuroscience, University of Siena, Via A. Moro 4, 53100 Siena, Italy; ^4^Department of Translational Research and New Technologies in Medicine and Surgery, Pisa University, 56100 Pisa, Italy

## Abstract

*Objective*. The aim of the study was to compare the pain symptoms of fibromyalgia patients exhibiting (FMS+PVD) and not exhibiting (FMS) comorbidity with provoked vulvodynia. *Study Design*. The case control study was performed in 39 patients who had been diagnosed with FMS and accepted to undergo gynaecological examination and in 36 healthy women (C). All patients completed standardized questionnaires for pain intensity, pain area, and psychological functioning. The gynaecological examination included vulvar pain pressure reactivity (Q-tip), pelvic tone assessment (Kegel manoeuver), and a semistructured interview collecting detailed information about pelvic symptoms and sexual function. *Results*. FMS+PVD patients displayed a higher number of associated symptoms than FMS patients. The vulvar excitability was significantly higher in FMS+PVD than in FMS and in both groups than in Controls. Half of FMS+PVD patients were positive to Kegel manoeuver and displayed higher scores in widespread pain intensity, STAI-Y2, and CESD levels than Kegel negative patients. *Conclusions*. The study reveals that increased vulvar pain excitability may occur in FMS patients independently of the presence of coital pain. Results suggest that coital pain develops in patients with higher FMS symptoms severity due to the cooperative effects of peripheral and central sensitization mechanisms.

## 1. Introduction

Fibromyalgia (FMS) is a condition of chronic widespread musculoskeletal pain characterized by steadily, diffuse, fluctuating musculoskeletal pain associated with hyperreactivity to deep pressure stimuli [1]. The chronic, widespread pain patients satisfying the 1990 criteria of the American College of Rheumatologists, that is, at least 11/18 positive tender points [2] and/or exhibiting higher symptoms severity [3], are classified as FMS. Fibromyalgia is often associated with regional pain syndromes characterized by central sensitization such as low back pain, osteoarthritis, migraine, temporomandibular pain, irritable bowel syndrome, and interstitial cystitis [4, 5].

A larger number of women suffer from FMS with respect to men; in addition, women report multiple pain locations, heavier disabilities, lower self esteem, and poorer coping capacity than men [6]. Several studies have shown that FMS painful areas may include tights, lower back, and the perineum; it is worth noting that these regions are involved in comfortably positioning oneself for sexual intercourse [6, 7]. Painful positioning, especially if associated with anxiety and depression, may elicit negative changes,such as decrease in sexual arousal, excitement, and climax capability [8–10]. On the other hand, genital pain at intercourse may be due to many pelvic conditions such as infections and dermatological, metabolic, autoimmune, or neoplastic diseases; it can also be due to a clinical disorder that has been identified as a unique entity, the “burning vulva syndrome,” or vulvovestibular syndrome [11]. The latter is classified into the subgroups of provoked (PVD), unprovoked, and mixed vulvodynia [12] and is diagnosed by excluding gynaecological and dermatological diseases.

Recently, comorbidity of PVD and FMS has been clearly assessed [13–15] and it has been suggested that vulvodynia could be considered as a localized expression of the chronic widespread pain syndrome [1, 14]. In this perspective, the aim of the present study was to investigate the subjective, psychophysical, and clinical pain symptoms of FMS patients suffering also with PVD.

## 2. Methods

### 2.1. Participants and Procedures

The clinical rheumatologist of the team (Giovanni Biasi) selected chronic widespread pain patients by a general screening examination and addressed them to the Psychophysics Laboratory of the Department of Medical and Surgical Sciences and Neuroscience to confirm the diagnosis of fibromyalgia. Patients completed a battery of psychophysical tests and self-administered questionnaires for the diagnosis of fibromyalgia according to ACR-90 criteria [2]. An epidemiological-anamnestic questionnaire, in use in our laboratory, provided the general picture of their disorders, and self-administered psychometric questionnaires, validated for *Italian *(CES-D [16] and STAI-Y2 [17]), assessed the psychological functioning.

Algometry was used to determine positive tender points number and threshold, while Von Frey hairs were used to determine punctuate superficial pain threshold; information about pain duration, present pain intensity (VAS, 0–100), and pain area and location [18] were collected [19]. The eighty consecutive patients whose diagnosis of fibromyalgia was confirmed were invited to complete their survey at the Sexual Medicine Clinics, Siena University Hospital. Thirty-nine out of 80 FMS patients showed up for the evaluation. All patients appeared free of gynaecological and dermatological conditions and of severe systemic and psychiatric illness, underwent a complete sexual evaluation, and were included in the study.

Thirty-six women with no malignancy, no acute or chronic pain symptoms, and no pelvic pathology or lower genital tract conditions who attended the outpatient unit of the Gynaecology Clinic (Siena University Hospital) for common care were recruited as a Control group (C) of vulvar excitability. They were matched with FMS patients for age, education, marital status, number of deliveries, and occurrence of menopause. Controls underwent a complete evaluation of sexual function and were free of pain areas at the manual gynaecological examination. They denied any localized and diffuse pain and, thus, were not submitted to quantitative sensory testing (tender points algometry, Von Frey). The study protocol is included in the clinical standard methods of investigation for the diagnosis of fibromyalgia in the Rheumatology Clinic, complies with the *Italian *current law, and has been approvedby the Scientific Committee of the Medical Faculty and by the Ethical Review Board of Siena University. The date of entry was June 2008 and the date of completion of data collection was January 2011. All participants signed an informed consent form.

The diagnosis of provoked vulvodynia (PVD) was reached (Anna Ghizzani) throughout the history of vulvar pain elicited by light pressure. Data on genital pain, its onset, and its occurrence with intercourse were collected through semistructured interviews; in both patients and Controls gynaecological examination consisted of naked eye observation of external genitalia to rule out dermatologic abnormalities and infections.

Q-tip pressure pain test evaluated vulvar reactivity and Kegel manoeuvre assessed the tonicity of pelvic floor muscles. The Q-tip test [20, 21], which has been recently validated for cutaneous allodynia [22, 23], is performed by gently touching the vestibule with a cotton-tipped stick at the 2, 4, 6, 8, and 10 o'clock positions. A constant, light pressure for approximately 5 seconds and the elicited sensation is rated by the subject on 6-point pain scale (VAS: 0: no pain—5: unbearable pain). We considered a site positive when the pain score ranged from 1 to 5. In healthy subjects, Q-tip stimulation applied in any area of the body, including the vulva, elicits a sensation of light touch [20, 21]. The number of positive sites, the positive sites mean pain intensity, and the sum of the positive sites scores were calculated for each patient.

All patients were examined bimanually to assess the tonicity of the muscles of the pelvic floor and specifically the pubococcygeal portion of the elevator ani (Kegel manoeuvre) [24]. Patients were instructed to squeeze the pubococcygeal muscles to their maximum against the examiner's fingers and to release them. The test is considered positive if a muscle spasm occurs and/or a difficulty in relaxing the muscle is detected.

The criteria for the diagnosis of PVD were (a) exclusion of vulvar infections, neoplastic diseases, vulvar pain of gynaecological, and dermatological origin; (b) presence of pain during intercourse and/or of painful sensations elicited by light pressure stimuli applied to the vestibule in the latest 3 months or longer.

On the basis of the above-mentioned criteria, participants (*n* = 75) were divided in fibromyalgic (FMS, *n* = 18), FMS with provoked vulvodynia (FMS + PVD, *n* = 21), and Controls (C, *n* = 36).

### 2.2. Statistical Analysis

SPSS.15 was used for MANOVAs of (a) demographical parameters, (b) questionnaires scores (STAI-Y2, CES-D), (c) pain parameters and psychophysical variables, and (d) vulvar punctate pain pressure scores (Q-tip). Intercourse frequency, frequency of associated symptoms, and occurrence of pelvic floor hypertonicity (Kegel manoeuvre) were assessed through Wilcoxon test. Significance was set at *P* < 0.05.

## 3. Results

### 3.1. Patients History

The mean age of patients was 47 ± 32 yrs; demographic characteristics (age, marital and employment status, education, and menopause) as well as anxiety and depression were similar in FMS and FMS + PVD patients.

PVD was frequently observed in the FMS patients (21/39 = 53.82%). The percentage of patients with associated symptoms and the percentage of symptoms per patient (pelvic/gynaecological/tights pain, osteoarthritis syndromes, chronic fatigue, peripheral neuropathies, and visceral and neoplastic diseases) were higher in FMS + PVD than in FMS patients (patients percentage per group, *Z* = 2.219, *P* < 0.026; symptoms percentage per patient, *Z* = 2.009, *P* = 0.053).

Only 25% of the FMS + PVD patients were aware of the PVD onset: in fact, vulvar pain could develop suddenly, gradually, at first intercourse, during pregnancy, or since childhood and could precede or follow the FMS onset. The remaining 75% of patients believed that their coital pain was just a corollary symptom of their FMS disease. All FMS + PVD patients, even those who had a clear understanding of the different nature of their genital pain, reported that their main concern had always been widespread pain.

Vulvar pain in FMS + PVD patients could be elicited by light touching as in foreplay causing them to avoid intercourse or, during sex, it could become excruciating to the point of forcing the couple to stop making love. Half of the patients of the FMS + PVD group and 95% of the FMS group were active once a month,while 20% of FMS + PVD had been abstinent during the last year or for a longer time. Postcoital pain was common in FMS + PVD (75% of patients) and lasted at least 1 hour in 30% of them and about 24 hours in 25% of them.

### 3.2. Pain Parameters and Quantitative Sensory Testing

Multivariate ANOVA did not reveal any significant difference between FMS and FMS + PVD patients in pain thresholds at positive tender points, number of positive tender points, pain duration, pain area, pain intensity, and Von Frey superficial punctate pain threshold (Table 1). The two groups did not differ for CES-D (mean ± sd. FMS + PVD: 23.43 ± 7.49; FMS: 22.88 ± 8.53) and STAI-Y2 (FMS + PVD: 47.43 ± 6.07; FMS: 45.07 ± 9.81).

### 3.3. Q-Tip Pain Pressure Test

The FMS + PVD, FMS, and C groups were significantly different for the number of positive Q-tip sites (*F*(2,73) = 65.192, *P* < 0.0001), the mean vulvar pain intensity (*F*(2,73) = 68.855, *P* < 0.0001), and the total pain intensity scores (*F*(2,73) = 48.792, *P* < 0.001).

**Figure 1:**
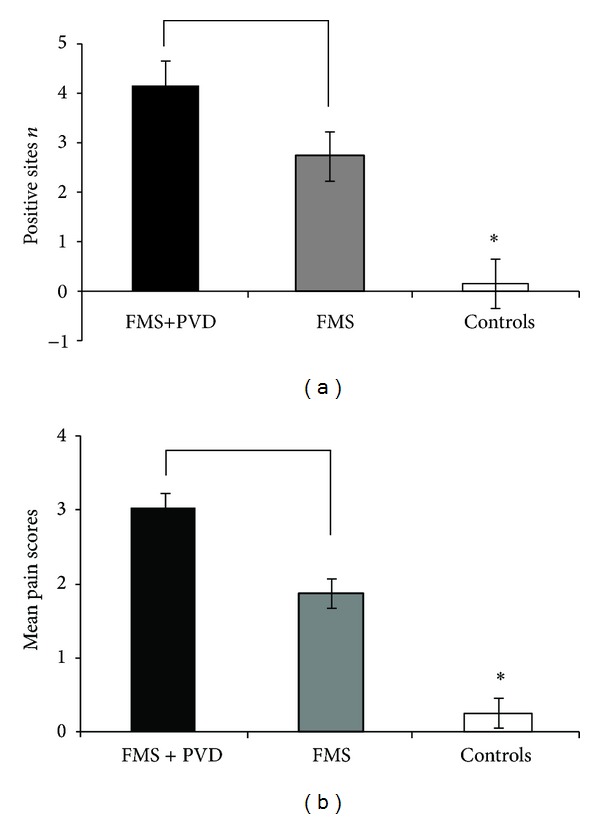
Vulvar pressure pain. (a) Mean values of positive sites numbers. (b) Mean Q-tip pain intensity scores (VAS = 0–5) in response to vulvar pressure stimuli. FMS: fibromyalgia patients; FMS + PVD: patients with fibromyalgia associated with provoked vulvodynia; C: Controls.

Figure 1(a) shows that the number of positive Q-tip sites was significantly different between FMS and FMS + PVD (*t*(1,37) = 2.446, *P* < 0.024), between FMS + PVD and Controls (*t*(1,55) = 16.145, *P* < 0.0001), and between FMS and Controls (*t*(1,52) = 6.962, *P* < 0.0001).

**Figure 2:**
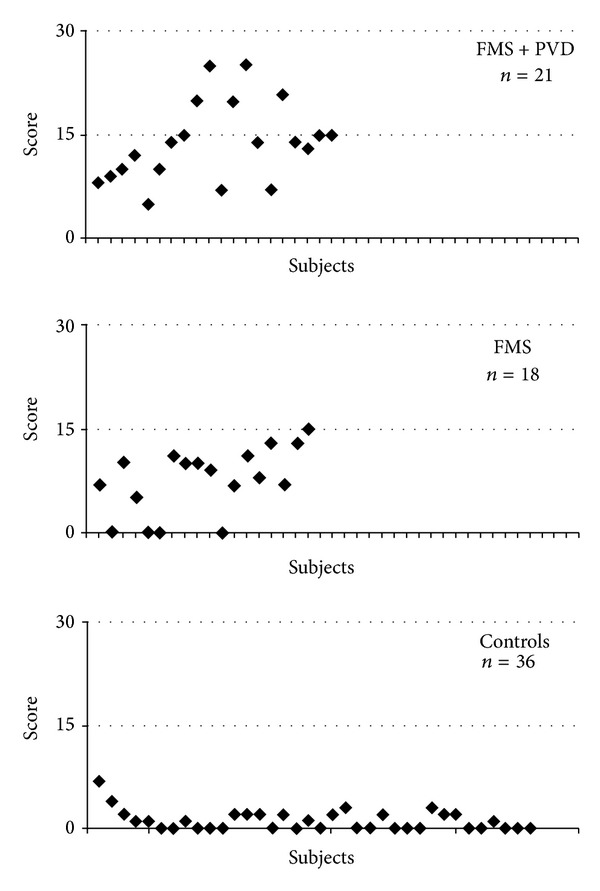
Distribution of total vulvar pain intensity scores in all groups. Note the overlap of FMS and FMS + PVD scores. Abbreviations as in Figure 1.

The mean Q-tip pain intensity score (Figure 1(b)) was significantly different between FMS and FMS + PVD (*t*(1,37) = 2.832, *P* < 0.008), FMS + PVD and Controls (*t*(1,55) = 10.599, *P* < 0.0001), and FMS and Controls (*t*(1,52) = 5.877, *P* < 0.001).  Figure 2 shows the distribution of the total pain intensity scores of the 3 groups (FMS + PVD > C, *t*(1,55) = 12.586, *P* < 0.0001); FMS > C, (*t*(1,52) = 7.394, *P* < 0.0001); FMS + PVD > FMS, *t*(1,37) = 3.666, *P* < 0.001).

### 3.4. Pelvic Floor Hypertonicity (Kegel Test)

**Figure 3:**
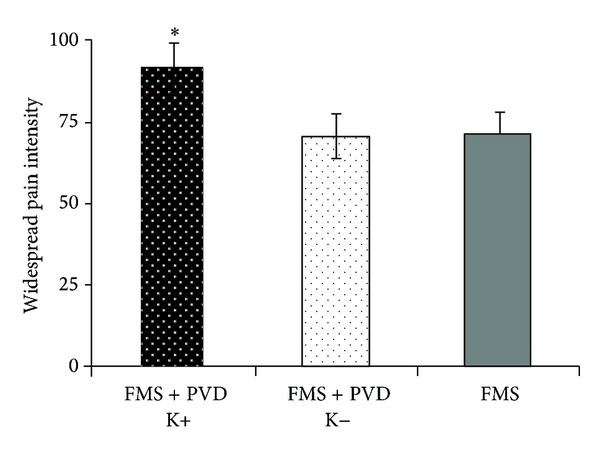
Widespread pain intensity. Pain scores (VAS = 0–100) in Kegel positive and Kegel negative FMS + PVD patients and in FMS patients.

Pelvic floor hypertonicity did not occur in C and FMS patients, but was present in 10 out of 21 FMS + PVD patients. Multivariate ANOVA on quantitative sensory testing values and questionnaires scores of FMS patients and FMS + PVD patients positive and negative to the Kegel manoeuvre revealed significant differences in widespread pain intensity (VAS (*F*(1,37) = 7.038, *P* < 0.013), STAI-Y2 (*F*(1,37) = 3.966, *P* < 0.034) and CES-D (*F*(1,37) = 3.879, *P* < 0.031). Widespread pain intensity (Figure 3) was significantly higher in Kegel positive FMS + PVD than in FMS patients (*F*(1,20) = 4.467, *P* < 0.047) and not significantly different between Kegel negative FMS + PVD and FMS patients. Questionnaires scores did not differ significantly between FMS and the two FMS + PVD subgroups. Within the FMS + PVD patients, the subgroup with pelvic floor hypertonicity was significantly different from the subgroup without pelvic floor hypertonicity (Figure 3). In fact, Kegel positive FMS + PVD patients exhibited higher widespread pain intensity (VAS, *t*(1,20) = 4.064, *P* < 0.0001) (Figure 3), anxiety (STAI-Y2, *t*(1,20) = 4.054, *P* < 0.002); Kegel positive (Mean ± SD): 54.17 ± 5.94; Kegel-negative: 41.30 ± 6.46), and depression levels (CES-D, *t*(1,20) = 2.546, *P* < 0.05; Kegel positive: 30.50 ± 13.87; Kegel negative: 15.75 ± 7.74).

## 4. Discussion

Our study shows that FMS + PVD patients display a larger number of associated symptoms than FMS patients and that most of the FMS patients exhibit increased vulvar pain excitability with respect to Controls.

Compared to FMS, FMS + PVD patients show higher frequency of pelvic muscle hypertonicity very often associated with vulvar pain [25–27]. FMS + PVD patients who are also affected by pelvic floor hypertonicity show higher levels of widespread pain intensity, anxiety, and depression than FMS + PVD without pelvic floor hypertonicity. It could be suggested that high levels of anxiety and depression may facilitate the development of PVD in FMS patients.

There is evidence that FMS patients may display different levels of sensitization and that the highest levels are observed in patients with multiple comorbidities [28]. Moreover, comorbid conditions independently associated with chronic pain increase the odds of reporting chronic pain in an additive manner [29].

Neuropathic mechanisms are involved in both PVD and FMS. Indeed, structural changes in vulvar innervation, consisting of sprouting between epithelial cells, increased in intraepithelial nerve endings and papillary TRPV1 afferent fibres, have been described in vulvodynia patients [30–32]. The vulvar allodynia that is elicited by mechanical and thermal (heat and cold) low threshold stimuli is probably associated with local peripheral sensitization of both polymodal C-mechanoheat nociceptors and normally mechanoinsensitive C-nociceptors [33–35]. Since these peripheral mechanisms are associated with abnormal temporal summation to pressure stimuli [34, 36] and allodynia is not restricted to the vulvar vestibule [34], the involvement of both peripheral and central sensitization mechanisms in the pathogenesis of vulvodynia has been suggested [34, 37]. This is supported by repeated observations that PVD is associated to generalized lowered thresholds to pressure and heat nociceptive stimuli [33–35].

There is also evidence that a peripheral neuropathic component, mainly neurogenic inflammation [38, 39], contributes to central sensitization in fibromyalgia [40, 41]. In FMS patients, microneurography studies have shown the occurrence of spontaneous activity, multiple spikes, abnormal sensitization, abnormalities in activity of in nociceptive fibers [42], and also frequent systemic symptoms pertaining to dysesthetic, evoked, paroxysmal, and thermal domains that are typical of neuropathic pain [43]. Thus, several types of peripheral inputs may contribute to develop different levels of central sensitization in FMS + PVD and in FMS.

It could be suggested that the vulvar hyperexcitability that we have described in most FMS patients probably represents the local expression of the generalized superficial allodynia [2, 19], as the vulvar vestibule displays a somatic innervation similar to that occurring in the skin [44, 45]. Therefore, it is not surprising that our FMS patients considered their provoked vulvodynia just as a component of widespread pain. Local nerve fibre proliferation has been reported in provoked vulvodynia [30, 31], but it has never been investigated in FMS. In these patients, vulvar hyperexcitability alone may be not sufficient to produce coital pain, but could contribute to enhance spatial pain summation. In fact, larger local areas of pain are associated with higher pain intensity [46–48].

Our findings confirm that FMS and PVD syndromes can develop independently and that patients affected by FMS + PVD display sexual disfunctions similar to those reported by patients affected only by PVD [12, 49, 50]. The reduction in the intercourse frequency in FMS + PVD compared to FMS may be due not only to coital pain but also to the greater symptom severity which influences these patients quality of life [6, 51].

A limitation of the study is that the patients' recruitment modality does not allow to infer the epidemiological consistency of the association between PVD and FMS.

In conclusion, our preliminary findings confirm the hypothesis that pain mechanisms of different origin can cooperate in worsening pain symptoms. In particular, they suggest that fibromyalgic allodynia increases the risk of coital pain.

## Figures and Tables

**Table 1:** Quantitative sensory testing.

	FMS + PVD		FMS	
	Mean	SD	Mean	SD
Pain duration (months)	113.53	95.44	104.88	151.48
Pain intensity (VAS: 0 100)	79.00	17.03	69.65	24.38
Pain area (body surface %)	56.85	23.21	60.59	17.11
Positive TP number	14.53	2.42	14.76	2.19
Positive TP threshold (kg)	2.43	0.55	2.61	0.38
Von Frey (gr)	350.03	281.30	521.20	290.82

## References

[1] Yunus MB (2001). Central sensitivity syndromes. A unified concept for fibromyalgia and other similar maladies. *Journal of Indian Rheumatologic Association*.

[2] Wolfe F, Smythe HA, Yunus MB (1990). The American College of Rheumatology 1990. Criteria for the classification of fibromyalgia. Report of the Multicenter Criteria Committee. *Arthritis and Rheumatism*.

[3] Wolfe F, Clauw DJ, Fitzcharles M-A (2010). The American College of Rheumatology preliminary diagnostic criteria for fibromyalgia and measurement of symptom severity. *Arthritis Care and Research*.

[4] Kindler LL, Bennett RM, Jones KD (2011). Central sensitivity syndromes: mounting pathophysiologic evidence to link fibromyalgia with other common chronic pain disorders. *Pain Management Nursing*.

[5] Williams DA, Clauw DJ (2009). Understanding fibromyalgia: lessons from the broader pain research community. *Journal of Pain*.

[6] Rosenbaum TY (2010). Musculoskeletal pain and sexual function in women. *Journal of Sexual Medicine*.

[7] Ryan S, Hill J, Thwaites C, Dawes P (2008). Assessing the effect of fibromyalgia on patients’ sexual activity. *Nursing Standard*.

[8] Kalichman L (2009). Association between fibromyalgia and sexual dysfunction in women. *Clinical Rheumatology*.

[9] Shaver JLF, Wilbur J, Robinson FP, Wang E, Buntin MS (2006). Women’s health issues with fibromyalgia syndrome. *Journal of Women’s Health*.

[10] Tikiz C, Muezzinoglu T, Pirildar T, Taskin EO, Firat A, Tuzun C (2005). Sexual dysfunction in female subjects with fibromyalgia. *Journal of Urology*.

[11] Friedrich EG (1987). Vulvar vestibulitis syndrome. *The Journal of Reproductive Medicine*.

[12] Moyal-Barracco M, Lynch PJ (2004). 2003 ISSVD terminology and classification of vulvodynia: a historical perspective. *The Journal of Reproductive Medicine*.

[13] Arnold LD, Bachmann GA, Rosen R, Kelly S, Rhoads GG (2006). Vulvodynia: characteristics and associations with comorbidities and quality of life. *Obstetrics and Gynecology*.

[14] White KP, Harth M (2001). Classification, epidemiology, and natural history of fibromyalgia. *Current Pain and Headache Reports*.

[15] Reed BD, Harlow SD, Sen A, Edwards RM, Chen D, Haefner HF (2012). Relationship between vulvodynia and chronic co-morbid conditions. *Obstetrics and Gynecology*.

[16] Fava CA, Canestrari R (1982). Versione italiana del CESD per la valutazione degli stati depressivi. *Nuovi Metodi di Psicometria*.

[17] Pedrabissi L, Santinello M (1989). Inventario per l'ansia di “stato“ e di “tratto”. *Nuova Versione Italiana dello STAY. Forma Y: Manuale*.

[18] Margolis RB, Tait RC, Krause SJ (1986). A rating system for use with patient pain drawings. *Pain*.

[19] Carli G, Suman AL, Biasi G, Marcolongo R (2002). Reactivity to superficial and deep stimuli in patients with chronic musculoskeletal pain. *Pain*.

[20] Haefner HK (2000). Critique of new gynecologic surgical procedures: surgery for vulvar vestibulitis. *Clinical Obstetrics and Gynecology*.

[21] Masheb RM, Lozano C, Richman S, Minkin MJ, Kerns RD (2004). On the reliability and validity of physician ratings for vulvodynia and the discriminant validity of its subtypes. *Pain Medicine*.

[22] Jarrell J (2009). Demonstration of cutaneous allodynia in association with chronic pelvic pain. *Journal of Visualized Experiments*.

[23] Yarnitsky DMG (2006). Neurophysiological examinations in neuropathic pain. Quantitative sensory testing. *Handbook of Clinical Neurology*.

[24] Kegel AH (1952). Sexual functions of the pubococcygeus muscle. *Western Journal of Surgery, Obstetrics, and Gynecology*.

[25] Glazer HI, Rodke G, Swencionis C, Hertz R, Young AW (1995). Treatment of vulvar vestibulitis syndrome with electromyographic biofeedback of pelvic floor musculature. *The Journal of Reproductive Medicine*.

[26] Jantos M, Glazer HI (1997). Establishing the diagnosis of vulvar vestibulitis. *Journal of Reproductive Medicine*.

[27] Jantos M (2008). Vulvodynia: a psychophysiological profile based on electromyographic assessment. *Applied Psychophysiology Biofeedback*.

[28] Affaitati G, Fabrizio A, Costa G Sensory asset in subgroups of fibromyalgia patients with different comorbidities.

[29] Dominick CH, Blyth FM, Nicholas MK (2012). Unpacking the burden: understanding the relationships between chronic pain and comorbidity in the general population. *Pain*.

[30] Weström LV, Willén R (1998). Vestibular nerve fiber proliferation in vulvar vestibulitis syndrome. *Obstetrics and Gynecology*.

[31] Tympanidis P, Terenghi G, Dowd P (2003). Increased innervation of the vulval vestibule in patients with vulvodynia. *British Journal of Dermatology*.

[32] Tympanidis P, Casula MA, Yiangou Y, Terenghi G, Dowd P, Anand P (2004). Increased vanilloid receptor VR1 innervation in vulvodynia. *European Journal of Pain*.

[33] Bohm-Starke N, Hilliges M, Brodda-Jansen G, Rylander E, Torebjörk E (2001). Psychophysical evidence of nociceptor sensitization in vulvar vestibulitis syndrome. *Pain*.

[34] Pukall CF, Binik YM, Khalifé S, Amsel R, Abbott FV (2002). Vestibular tactile and pain thresholds in women with vulvar vestibulitis syndrome. *Pain*.

[35] Granot M, Friedman M, Yarnitsky D, Zimmer EZ (2002). Enhancement of the perception of systemic pain in women with vulvar vestibulitis. *British Journal of Obstetrics and Gynecology*.

[36] Lowenstein L, Vardi Y, Deutsch M (2004). Vulvar vestibulitis severity—assessment by sensory and pain testing modalities. *Pain*.

[37] Yunus MB (2012). The prevalence of fibromyalgia in other chronic pain conditions. *Pain Research and Treatment*.

[38] Kim S-H, Jang TJ, Moon IS (2006). Increased expression of N-methyl-D-aspartate receptor subunit 2D in the skin of patients with fibromyalgia. *Journal of Rheumatology*.

[39] Littlejohn GO, Weinstein C, Helme RD (1987). Increased neurogenic inflammation in fibrositis syndrome. *Journal of Rheumatology*.

[40] Dworkin RH, Fields HL (2005). Introduction: fibromyalgia from the perspective of neuropathic pain. *Journal of Rheumatology*.

[41] Kim S-H (2007). Skin biopsy findings: implications for the pathophysiology of fibromyalgia. *Medical Hypotheses*.

[42] Solà R, Collado A, Antonelli F, Serra J Is fibromyalgia a special type of small fibers neuropathy? A microneurography study.

[43] Martinez-Lavin M (2006). Fibromyalgia is a neuropathic pain syndrome. *Journal of Rheumatology*.

[44] Cervero F (1994). Sensory innervation of the viscera: peripheral basis of visceral pain. *Physiological Reviews*.

[45] Friedrich EG (1983). The vulvar vestibule. *The Journal of Reproductive Medicine*.

[46] Staud R, Price DD, Robinson ME, Vierck CJ (2004). Body pain area and pain-related negative affect predict clinical pain intensity in patients with fibromyalgia. *Journal of Pain*.

[47] Staud R, Vierck CJ, Cannon RL, Mauderli AP, Price DD (2001). Abnormal sensitization and temporal summation of second pain (wind-up) in patients with fibromyalgia syndrome. *Pain*.

[48] Vierck CJ (2006). Mechanisms underlying development of spatially distributed chronic pain (fibromyalgia). *Pain*.

[49] Gordon AS, Panahian-Jand M, McComb F, Melegari C, Sharp S (2003). Characteristics of women with vulvar pain disorders: responses to a web-based survey. *Journal of Sex & Marital Therapy*.

[50] Bachmann GA, Rosen R, Finn VW (2006). Vulvodynia: a state-of-the-art consensus on definitions, diagnosis and management. *The Journal of Reproductive Medicine*.

[51] Croft P, Burt J, Schollum J, Thomas E, Macfarlane G, Silman A (1996). More pain, more tender points: is fibromyalgia just one end of a continuous spectrum?. *Annals of the Rheumatic Diseases*.

